# Parasympathetic Responses to Face Cooling in Adolescents with Sport-Related Concussion and After Clinical Recovery

**DOI:** 10.1089/neur.2024.0138

**Published:** 2025-01-23

**Authors:** Mohammad N. Haider, Haley M. Chizuk, Blair D. Johnson, Joel S. Burma, Jaffer A. Sayeed, Emma Anderson, Barry S. Willer, John J. Leddy

**Affiliations:** ^1^ Department of Orthopaedics, Jacobs School of Medicine and Biomedical Sciences, State University of New York at Buffalo, Buffalo, New York, USA.; ^2^ Department of Kinesiology, School of Public Health, Indiana University Bloomington, Bloomington, Indiana, USA.; ^3^ Faculty of Kinesiology, University of Calgary, Alberta, Canada.; ^4^ Jacobs School of Medicine and Biomedical Sciences, State University of New York at Buffalo, Buffalo, New York, USA.; ^5^ Department of Psychiatry, Jacobs School of Medicine and Biomedical Sciences, State University of New York at Buffalo, Buffalo, New York, USA.

**Keywords:** autonomic nervous system, face cooling, heart rate variability, sport-related concussion

## Abstract

Face cooling (FC) initiates the mammalian dive reflex, which elicits a parasympathetic autonomic response. In our pilot study, collegiate athletes had a blunted parasympathetic response to FC within 10 days of sport-related concussion (SRC). The objective of the current study was to assess the FC response in adolescent athletes with acute SRC and after clinical recovery. Symptomatic adolescents with SRC (*n* = 23, 15.48 ± 1.2 years, 52% male) had heart rate (HR) and blood pressure (BP) measured during the FC test (7.83 ± 2.5 days since injury) and again after clinical recovery (46.44 ± 36.4 days later). Controls (*n* = 24, 15.83 ± 1.6 years, 58% male) performed the same assessments twice (48.00 ± 18.9 days apart). The main outcome measures were the rate of change in HR and HR variability (HRV) during the first 2 min of FC. Throughout FC, we found no significant differences between groups at the initial visit in the rate of change for HR (mean difference = 2.58 [−0.33, 5.50] bpm/min, *p* = 0.082), mean arterial BP (−0.02 [−3.49, 3.45] mmHg/min, *p* = 0.990), root mean square of successive differences (−13.46 [−34.02, 7.10] ms/min, *p* = 0.197) or low to high-frequency ratio (0.24 [−0.77, 1.25], *p* = 0.637). We also found no differences in our main outcome measures among concussed adolescents with delayed recovery (*n* = 10) compared with those with normal recovery (*n* = 13). A history of prior concussion had a significant effect on the HR and HRV responses to FC, suggesting that SRC may have prolonged effects on the autonomic nervous system (ANS). We conclude that acutely concussed adolescents do not differ from controls in parasympathetic response to FC acutely or upon recovery but that a history of concussion affects this response. We recommend that future studies control for concussion history when investigating the ANS in concussed adolescents.

## Introduction

Sport-related concussion (SRC) is a public health concern.^1,2^ SRCs are caused by blunt trauma to the head or elsewhere on the body with force transmitted to the brain, resulting in transient neurological dysfunction.^2,3^ The typical duration of SRC in adolescents is less than 1 month.^2^ However, ∼30% of patients take longer to recover, known as persisting postconcussive symptoms (PPCS).^4^ The signs and symptoms of SRC are diverse, reflecting a functional disturbance that is not seen in standard brain imaging studies.^3^ The literature suggests that some symptoms of concussion, such as exercise intolerance,^5,6^ sleep disturbance,^7,8^ and vestibulo-ocular impairments,^9^ may be caused by altered autonomic nervous system (ANS) function.^10^ One measure of ANS function is heart rate (HR) variability (HRV), the consistency of the timing between consecutive heartbeats.^11^ Athletes with SRC demonstrate altered HRV at rest and during exercise.^12^ It has been hypothesized that this reflects a functional uncoupling of central ANS control of cardiovascular function.^13^

Face cooling (FC), that is, cooling the forehead, eyes, and cheeks, triggers the mammalian diving reflex.^14^ This causes the trigeminal nerve to evoke a transient (∼1–2 min) increase in cardiac parasympathetic activity followed by sympathetically mediated increased blood pressure (BP).^14^ In a pilot case-control study,^15^ concussed collegiate athletes within 10 days of injury had a blunted parasympathetic response to FC when compared with controls who did not have a concussion within the past year. Concussed athletes also demonstrated lower sympathetically mediated increases in BP during FC. It was concluded that the parasympathetic and sympathetic branches of the ANS demonstrated a blunted response to FC.^15^ Whether the altered autonomic response after SRC normalized upon clinical recovery was not determined.

The primary aim of this study was to evaluate the FC response in adolescent athletes with SRC while they were symptomatic and after clinical recovery. Prolonged recovery (i.e., development of PPCS)^16^ and a history of prior concussion,^17^ have been identified as potential confounders in studies of ANS function. Therefore, we also studied their effect on the FC response. The secondary aim of this study was to determine if the HR and HRV responses to FC were reliable in adolescents because prior reliability studies have been performed only in adults.^17^ We hypothesized that adolescents with SRC would have a blunted response to FC that normalized upon clinical recovery and that the HR and HRV responses to FC would be reliable over multiple assessments in healthy adolescents.

## Methods

### Study design

The University at Buffalo’s institutional review board (IRB) approved this prospective case-control study. Between October 2016 and February 2020, adolescents diagnosed with SRC at three university-affiliated sports medicine clinics in Buffalo, NY, were asked to participate in the study. A research assistant explained the study and obtained written consent in a compliant setting. Parental consent and participant assent were obtained for all minors (aged 13–17). Participants came to the physiology lab within 10 days of injury (Visit 1) and returned within 2 weeks of successful completion of a return-to-play (RTP) protocol (Visit 2).^2^ Medical clearance from a physician was required before participants initiated RTP protocols. Participants completed their initial research visit and followed up with their physicians in accordance with international clinical guidelines.^2,16^

Snowball sampling was used to recruit controls by asking participants with SRC if they had a teammate who might be interested in participating in the study. This sampling method was used because the physiological parameters of interest are associated with cardiovascular fitness; therefore, controls needed to be in at least one organized sport.^18^ Controls repeated the FC test 4–6 weeks after their first visit to parallel the usual recovery time of 1 month for adolescents with SRC.^2^

### Participants

The inclusion criteria for concussed participants were: (1) age 13–18 years; (2) diagnosed with an SRC by a relevant clinician; and (3) injury occurred within 10 days of initial research assessment. The exclusion criteria for concussed participants were: (1) a current or prior traumatic brain injury more severe injury than a concussion; (2) more than 3 prior concussions; (3) active substance abuse/dependence; and (4) currently taking medications that affect the ANS (e.g., attention disorder stimulants and mood stabilizers). The inclusion criteria for controls were: (1) age 13–18 years; (2) no history of concussion within the past year; and (3) actively participating in at least one organized sport. Exclusion criteria for controls were identical to those for participants with concussions.

### Study definitions and standard treatment protocols

#### Diagnosis of Concussion

SRC was diagnosed using recent international guidelines,^2^ including (1) onset of concussion-like symptoms,^19^ associated with a head injury or injury to another part of the body with force transmitted to the brain; (2) impairments on a concussion-focused clinical examination,^20^ performed by an experienced sports medicine physician; and (3) exercise intolerance on graded exertion testing.^21^

#### Management of Concussion

After diagnosis, concussed participants were instructed not to participate in sports or recreational activities that may result in another head injury and to socialize, study, and work at levels that did not exacerbate their symptoms.^22^ Concussed participants were assessed weekly by a physician who determined clinical recovery. Participants who did not recover by 4 weeks received multidisciplinary care that may have included vestibulo-ocular therapy, aerobic exercise treatment, vision therapy, and/or cognitive behavioral therapy for mood-related symptoms.^23^

#### Recovery criteria

Medical recovery was defined as: (1) a resolution of symptoms to baseline; (2) a concussion-focused physical examination within normal limits; and (3) an ability to exercise to exhaustion without exacerbating concussion-like symptoms.^24^ After recovery, participants began an RTP protocol under the supervision of their school or sports team athletic trainer.^2^

### Equipment

#### Data acquisition

BIOPAC Systems, Inc., MP160 system was used to acquire and analyze physiological data. The MP160 is a 16-channel core system with a high-level transducer interface module that uses AcqKnowledge 5.0 software for visualization and processing.

#### Electrocardiogram

Bionomadix 3-lead electrocardiogram (EKG; DA100C, Biopac Systems, Goleta, CA) was used to obtain the R-R interval (RRI) for HRV analysis. HRV analysis was performed using Kubios HRV Software 5.0, which has built-in tools for EKG clean-up, including a QRS detector, beat-to-beat analysis, and R-wave correction.

#### Photo-plethysmograph

ClearSight photo-plethysmograph by Edwards Lifescience Inc., Irvine, CA (formerly Nexfin BMEYE, Amsterdam, Netherlands) was used on the left index finger (second metacarpal region) to obtain mean HR, mean arterial pressure (MAP), and cardiac output (CO).^25^ Stroke volume (SV) was calculated by dividing the CO by HR. As a quality control measure, a calibration using the Physiocal^™^ vascular unloading algorithm was used before each physiological test and turned off during the assessment.

### Experimental procedures and main outcome variables

Participants were instructed to refrain from alcohol, caffeine, and exercise for 12 h and food for 2 h before their visit. Participants were instrumented with a 3‐lead EKG and rested supine for 10 min in a quiet environment prior to FC. FC was performed by placing a pliable plastic bag filled with 2.5 L of ice water (∼0°C) on the forehead, eyes, and cheeks for 3 min. Room temperature (20–23°C) and humidity (15–25%) were controlled. Participants were informed that they could voluntarily stop the test at any time if they could not continue due to discomfort or for any other reason. The complete FC protocol has been published.^15^

EKG waveforms were visually inspected throughout data collection. The first 5 min of supine rest data were discarded, and baseline values were calculated as the mean of 6 min through 10. HR, RRI, and root mean square of the successive differences (RMSSD) of RRI,^11^ were derived from the time domain. Low-frequency (LF) and high-frequency (HF) power and the ratio of LF to HF (LF/HF ratio) were derived from the frequency domain using Fast Fourier transformation.^26^ HR, MAP, RMSSD, and LF/HF ratio were identified as the main outcome variables based upon a prior study,^15^ where HR and MAP were hemodynamic parameters, RMSSD corresponded to parasympathetic tone, and LF/HF ratio corresponded to sympathetic tone.

### Sample size estimation

The sample size was based on comparing the 2-min change from baseline in RMSSD. Our pilot study,^15^ found a mean change of 75 ± 105 ms^−2^ in 10 collegiate athletes and −10 ± 159 ms^−2^ in 11 concussed athletes. To find significant differences at an alpha of 0.05 and a beta of 0.80 using an independent samples *t-*test, we would require 41 participants in each group. This is discussed more in the transparency, rigor, and reproducibility summary.

### Statistical analyses

Independent samples *t-*test and χ^2^-test were used to assess demographic differences between groups. Mean values for each main outcome variable (HR, RRI, RMSSD, LF, HF, LF/HF, MAP, CO, and SV) were calculated at baseline and during each minute of FC. Baseline values for main outcome variables were subtracted from their subsequent values during each minute of FC to determine change. Groups were compared using Anovas. If significant, pairwise comparisons were performed using Tukey’s correction. Changes in main outcome variables (HR, MAP, RMSSD, and LF/HF) during FC were plotted with 95% confidence intervals (CI) and were compared using a mixed model linear regression. The main effects were FC minute (0–2), concussion (yes/no), and the interaction term of concussion * FC minute for Visits 1 and 2 separately. An autoregressive covariance pattern was identified, and a subject-specific random effects term was included in the model. Only the first 2 minutes of FC were analyzed since the sympathetic nervous system engages by the third minute.^15,27^ An analysis for confounding variables was performed using Visit 1 data. Concussed participants were stratified by PPCS (recovery time >28 days since injury) incidence and the changes in main outcome variables were compared. A similar model to compare concussion history was made. Last, to assess how reliable the response to FC is: an intraclass correlation coefficient (ICC) using a two-way mixed effects model between visits for concussed participants with normal recovery, concussed participants with delayed recovery (PPCS), and controls was calculated to assess the retest reliability of the FC response for the main outcome variables. A *p* value of 0.05 was defined as significant and all statistical analyses were performed using SPSS Version 29 (IBM Corp, Armonk, NY).

## Results

This trial was interrupted by the COVID-19 pandemic; hence, we were not able to meet our desired sample size. This is explained further in the transparency, rigor, and reproducibility section. Ninety-nine eligible concussed adolescents were seen during the enrollment period. Thirty-five adolescents were interested in participating, but six participants were unable to schedule a visit within 10 days of injury and were lost to follow-up. The analysis does not include data from three participants who were unable to complete 2 min of FC (15.3 ± 1.7 years, 2/3 males, 7.3 ± 1.7 days since injury, mean recovery time = 20.0 ± 6 days). In addition, data from three participants (15.0 ± 1.0 years, 2/3 males, and 4.3 ± 0.6 days since injury, mean recovery time = 25.7 ± 17.6 days) were not included in the analysis because their EKG recordings had multiple artifacts (>5% of raw data) identified during data processing. Hence, 23 concussed adolescents were included in the analysis. Twenty-seven nonconcussed adolescents agreed to participate, but one was lost to follow-up due to scheduling conflicts. Two participants (14.5 ± 0.5 years, both male) had EKG data with multiple artifacts that were removed from the analysis. Hence, 24 controls were included in the analysis. Groupwise demographics are presented in Table 1. No significant differences were seen at baseline except that the concussed participants reported more prior concussions than controls.

**Table 1.** Groupwise Demographics and Clinical Characteristics of Included Participants

	*Concussion* *n* = 23	*Control* *n* = 24	p
Age in years, mean ± SD	15.48 ± 1.16	15.83 ± 1.55	0.381
Sex, *n* (%) male	12 (52%)	14 (58%)	0.671
Height in meters, mean ± SD	1.67 ± 0.12	1.70 ± 0.08	0.362
Weight in kg, mean ± SD	69.51 ± 12.70	66.72 ± 11.99	0.443
History of concussion, *n* (%)			**<0.001**
0	7 (30.4%)	22 (91.7%)	
1	13 (56.5%)	0 (0%)	
2	1 (4.3%)	1 (4.2%)	
3	2 (8.7%)	1 (4.2%)	
Symptom severity, mean ± SD	35.79 ± 21.97	—	—
Days since injury to Visit 1, mean ± SD	7.83 ± 2.46	—	—
Recovery time since injury, mean ± SD	30.48 ± 22.02	—	—
Days between research visits, mean ± SD	46.44 ± 36.39	48.00 ± 18.93	0.871
Incidence of PPCS, *n* (%)	10 (44%)	—	—

Bold values indicate significant differences.

kg, kilogram; PPCS, persisting postconcussion symptoms; SD, standard deviation.

Table 2 presents the mean absolute values of all variables at baseline, 2 min of FC, and during the first 2 min of FC. No differences were seen between groups on Anova at baseline or by 2 min except for the LF/HF ratio, which was not significant on pairwise comparisons. When assessing the change in main outcome variables over time, there was a smaller reduction in HR in concussed participants compared with controls at Visit 2 and a smaller reduction in CO in concussed participants compared with controls at Visit 1.

**Table 2.** Groupwise Cardiovascular Parameters at Baseline, Minute 2, and 2-Minute Change During Face Cooling

	Concussion V1	Concussion V2	Control V1	Control V2	*p* ^a^	*p* ^b^	*p* ^c^	*p* ^d^	*p* ^e^
Baseline									
HR in bpm	67.65 ± 9.94	74.38 ± 12.02	75.09 ± 13.20	73.97 ± 14.61	0.243	—	—	—	—
RRI in ms	898.75 ± 143.22	861.50 ± 146.82	817.90 ± 123.08	824.28 ± 121.08	0.173	—	—	—	—
RMSSD in m*s*	72.72 ± 51.08	82.10 ± 67.59	60.45 ± 45.13	62.02 ± 44.64	0.516	—	—	—	—
LF FFT in ms^−2^	1775.01 ± 2756.68	2314.30 ± 3172.49	1467.40 ± 1998.78	1066.69 ± 1286.39	0.405	—	—	—	—
HF FFT in ms^−2^	3052.96 ± 4838.59	3981.49 ± 6474.50	1888.11 ± 2790.09	1704.08 ± 2396.13	0.309	—	—	—	—
LF/HF ratio	1.76 ± 2.34	1.42 ± 2.46	2.18 ± 4.64	1.02 ± 1.03	0.617	—	—	—	—
SV in cm^3^	106.91 ± 17.85	102.48 ± 18.86	95.90 ± 25.23	102.78 ± 21.70	0.812	—	—	—	—
CO in L/min	7.22 ± 1.11	7.56 ± 1.26	7.25 ± 2.37	7.57 ± 1.74	0.551	—	—	—	—
MAP in mmHg	87.30 ± 6.41	84.20 ± 11.25	81.90 ± 13.47	85.11 ± 10.34	0.885	—	—	—	—
Minute 2									
HR in bpm	67.79 ± 9.50	70.81 ± 13.98	66.86 ± 9.51	66.57 ± 12.49	0.234	—	—	—	—
RRI in ms	935.45 ± 130.77	868.32 ± 199.03	920.28 ± 159.81	951.46 ± 196.54	0.456	—	—	—	—
RMSSD in ms	117.60 ± 83.59	107.65 ± 83.54	129.74 ± 85.41	125.64 ± 95.21	0.854	—	—	—	—
LF FFT in ms^−2^	3175.83 ± 2940.89	4211.27 ± 5966.11	2839.14 ± 2923.39	2943.19 ± 3413.69	0.675	—	—	—	—
HF FFT in ms^−2^	5760.64 ± 6532.42	5451.11 ± 7941.60	8363.90 ± 8833.80	5985.97 ± 6719.77	0.574	—	—	—	—
LF/HF ratio	0.74 ± 0.39	1.33 ± 1.11	0.69 ± 0.74	0.75 ± 0.65	**0.029**	0.996	0.075	0.994	0.081
SV in cm^3^	108.49 ± 19.16	105.76 ± 20.58	97.68 ± 19.60	103.99 ± 18.58	0.323	—	—	—	—
CO in L/min	7.32 ± 1.21	7.42 ± 1.47	6.59 ± 1.58	6.99 ± 1.78	0.284	—	—	—	—
MAP in mmHg	99.53 ± 9.10	96.92 ± 12.24	96.33 ± 12.21	100.03 ± 11.05	0.645	—	—	—	—
Change from Baseline to Minute 2									
HR in bpm	−2.83 ± 11.34	0.41 ± 9.45	−7.96 ± 10.77	−8.80 ± 9.12	**0.014**	0.360	0.741	0.993	**0.026**
RRI in ms	33.06 ± 164.80	6.82 ± 139.76	102.38 ± 143.43	127.18 ± 143.47	**0.031**	0.423	0.942	0.947	0.053
RMSSD in ms	42.72 ± 78.26	25.55 ± 104.85	69.29 ± 79.88	63.62 ± 77.93	0.338	—	—	—	—
LF FFT in ms^−2^	1333.96 ± 3747.00	1896.97 ± 6732.03	1371.74 ± 3552.42	1876.50 ± 3253.78	0.961	—	—	—	—
HF FFT in ms^−2^	2578.28 ± 5608.33	1469.62 ± 10709.86	6475.79 ± 9139.03	4281.89 ± 6447.22	0.222	—	—	—	—
LF/HF ratio	−1.05 ± 2.41	−0.10 ± 2.74	−1.49 ± 4.71	−0.27 ± 1.19	0.415	—	—	—	—
SV in cm^3^	2.17 ± 9.03	1.56 ± 8.32	−0.26 ± 6.29	−1.85 ± 8.36	0.387	—	—	—	—
CO in L/min	0.18 ± 1.22	0.03 ± 1.23	−0.80 ± 1.20	−0.83 ± 1.02	**0.008**	**0.040**	0.973	>0.999	0.109
MAP in mmH*g*	16.33 ± 9.11	14.95 ± 7.76	16.00 ± 8.00	16.83 ± 7.15	0.901	—	—	—	—

Bold values indicate a significant finding.

^a^ *p*-value from one-way Anova.

^b^ *p*-value comparing concussion Visit 1 to control Visit 1.

^c^ *p*-value comparing concussion Visit 1 to Visit 2.

^d^ *p*-value comparing control Visit 1 to Visit 2.

^e^ *p*-value comparing concussion Visit 2 to Control Visit 2.

bpm, beats per minute; CO, cardiac output; cm, centimeter; FFT, fast fourier transformation; HF, high frequency; HR, heart rate; LF, low frequency; L/min, liters per minute; MAP, mean arterial pressure; mmHg, millimeters mercury; ms, millisecond; RRI, R-R interval; RMSSD, root mean square of successive differences; SV, stroke volume.

**FIG. 1.**
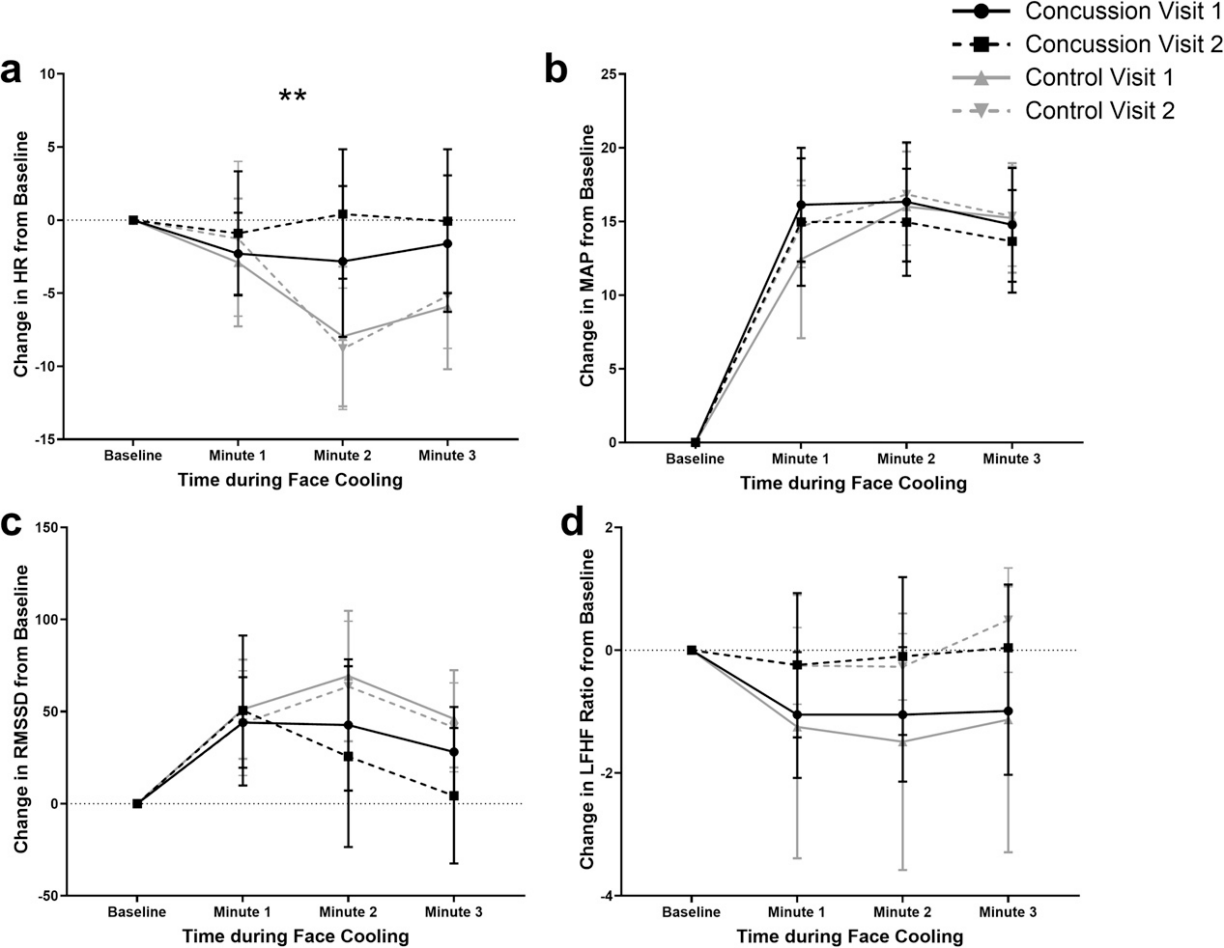
Groupwise means for **(a)** ΔHR, **(b)** ΔMAP, **(c)** ΔRMSSD, and **(d)** ΔLF/HF ratio during FC. **A significant difference between concussion and control over time during FC at Visit 2. FC, face cooling; HF, high frequency; HR, heart rate; LF, low frequency; MAP, mean arterial pressure; RMSSD, root mean square of the successive differences.

Figure 1a–d presents the groupwise means for ΔHR, ΔMAP, ΔRMSSD, and ΔLF/HF ratio during FC. Table 3 presents the results of the linear regression comparing change from baseline. The main effect of time during FC (FC minute) was significant for all variables except for LF/HF at Visit 2. The only significant difference between groups over time was that during the first 2 min of FC, concussed participants had a smaller change in HR by 4.59 (1.13, 8.04) bpm per minute compared with controls at Visit 2. The comparison of the change in HR over time between concussed and control participants at Visit 1 trended toward but did not reach statistical significance (*p* = 0.083), where concussed participants had smaller changes in HR. Parasympathetic tone (i.e., the RMSSD response to FC) was not significantly different between groups at Visit 1 (*p* = 0.197) or at Visit 2 (*p* = 0.178).

**Table 3.** Results of Linear Regression Predicting Change from Baseline over the First 2 Minutes of FC Between Concussion and Control Group

	Random effects	FC minute	Concussion	Concussion * FC minute
Visit 1, parameter estimate (CI) and *p* value				
HR in bpm	4.19 (−30.28, 38.67) *p* > 0.999	−3.98 (−6.02, −1.94) *p* < 0.001	−2.99 (−9.77, 3.80) *p* = 0.386	2.58 (−0.33, 5.50) *p* = 0.082
MAP in mmHg	**−7.00 (−12.33, −1.67)** ***p* = 0.010**	**8.19 (5.72, 10.66)** *p* < 0.001	1.14 (−6.43, 8.71) *p* = 0.766	−0.02 (−3.49, 3.45) *p* = 0.990
RMSSD in ms	−32.56 (−263.5, 200.4) *p* > 0.999	**34.70 (20.30, 49.09)** *p* < 0.001	14.82 (−33.50, 63.14) *p* = 0.545	−13.46 (−34.02, 7.10) *p* = 0.197
LF/HF ratio	0.66 (−16.75, 18.08) *p* > 0.999	**−0.75 (−1.45, −0.04)** ***p* = 0.039**	−0.25 (−2.70, 2.20) *p* = 0.839	0.24 (−0.77, 1.25) *p* = 0.637
Visit 2, parameter estimate (CI) and *p* value				
HR in bpm	5.27 (−23.11, 33.65) *p* > 0.999	**−4.38 (−6.78, −1.98)** *p* < 0.001	−5.81 (−13.38, 1.76) *p* = 0.131	**4.59 (1.13, 8.04)** ***p* = 0.010**
MAP in mmHg	**−6.62 (−11.40, −1.84)** ***p* = 0.007**	**8.49 (6.29, 10.68)** *p* < 0.001	1.41 (−5.34, 8.15) *p* = 0.680	−1.01 (−4.10, 2.08) *p* = 0.517
RMSSD in ms	−29.05 (−108.0, 49.87) *p* = 0.470	**31.81 (12.37, 51.25)** ***p* = 0.002**	25.05 (−38.84, 88.94) *p* = 0.439	−19.04 (−49.87, 8.80) *p* = 0.178
LF/HF ratio	0.11–2.11, 2.33) *p* = 0.921	−0.14 (−0.56, 0.29) *p* = 0.526	−0.10 (−1.55, 1.34) *p* = 0.890	0.09 (−0.52, 0.67) *p* = 0.776

Parameter estimates with 95% confidence intervals; the main fixed effect of FC minute is the rate of increase per minute in the entire sample; the main fixed effect of concussion is the mean difference between groups and is calculated as concussion minus control values; the interaction term of concussion * FC minute is the mean difference in the rate of change per minute between groups (Concussion minus control).

bpm, beats per minute; CI, confidence interval; FC, face cooling; HF, high frequency; HR, heart rate; LF, low frequency; MAP, mean arterial pressure; mmHg, millimeters mercury; ms, millisecond; RMSSD, root mean square of successive differences.

**FIG. 2.**
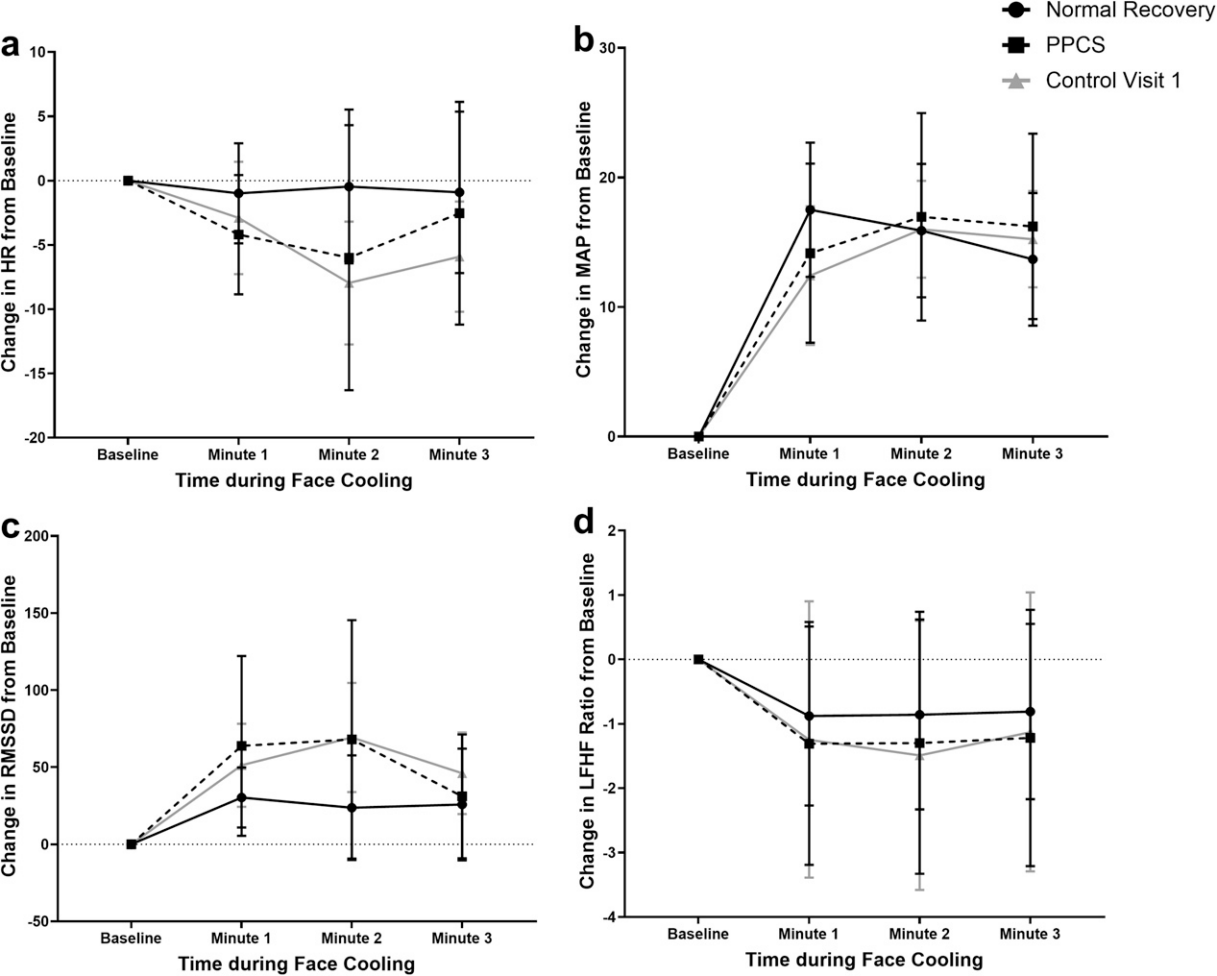
Groupwise means for ΔHR, ΔMAP, ΔRMSSD, and ΔLF/HF ratio during FC at Visit 1 stratified by normal recovery and PPCS. FC, face cooling; HF, high frequency; HR, heart rate; LF, low frequency; MAP, mean arterial pressure; PPCS, persisting postconcussive symptoms; RMSSD, root mean square of the successive differences.

Figure 2a–d presents the groupwise means for ΔHR, ΔMAP, ΔRMSSD, and ΔLF/HF ratio during FC at Visit 1 after stratifying the concussed participants by those who had normal recovery (*n* = 13) and those who had PPCS (*n* = 10). Controls at Visit 1 have been added to corresponding figures for reference but are not included in the analysis. Table 4 presents the *F*-statistics and *p* values of the exploratory regression model controlling for the incidence of PPCS. No significant differences were seen. Supplementary Figure S1 presents the groupwise means for ΔHR, ΔMAP, ΔRMSSD, and ΔLF/HF ratio during FC at Visit 2. Concussed participants who had a normal recovery were seen 30.4 ± 21.2 days after injury, while those who developed PPCS were 62.4 ± 42.3 days after injury. Controls have been added for reference in corresponding figures but are not included in the analysis. No significant differences were seen between concussed adolescents who had normal recovery or PPCS (PPCS * FC time predicting HR *p* = 0.429; MAP *p* = 0.922; RMSSD *p* = 0.328; and LFHF ratio *p* = 0.531). On visual inspection of the change in RMSSD (Supplementary Fig. S1), the normal recovery group was similar to controls at Visit 2. However, participants with PPCS had an exaggerated change in RMSSD during the first minute that fell below resting values at 3 min.

**Table 4.** Results of Exploratory Linear Regression Predicting Change in Main Outcome Variables at Visit 1 for Delayed Recovery and History of Concussion

Delayed recovery, F-statistic (p value)						
	FC minute		PPCS	PPCS * FC minute		
HR	2.961 (*p* = 0.091)		0.357 (*p* = 0.552)	2.235 (*p* = 0.141)		
MAP	**43.902** (*p* < 0.001)		0.087 (*p* = 0.769)	0.046 (*p* = 0.831)		
RMSSD	**10.700** (*p* = 0.002)		0.360 (*p* = 0.551)	2.519 (*p* = 0.118)		
LF/HF ratio	**4.757** (*p* = 0.033)		0.036 (*p* = 0.851)	0.273 (*p* = 0.603)		

History of concussion, *F*-statistic (*p* value)						
History of concussion, *F*-statistic (*p* value)	FC minute	Concussion * FC minute		History of concussion * FC minute	Concussion * history of concussion * FC minute	
HR	**4.142 (*p* = 0.044)**	0.013 (*p* = 0.910)		0.965 (*p* = 0.329)	**8.392 (*p* = 0.005)**	
MAP	**59.282 (***p* < 0.001)	0.225 (*p* = 0.637)		0.039 (*p* = 0.845)	0.012 (*p* = 0.915)	
RMSSD	**9.554 (*p* = 0.002)**	1.284 (*p* = 0.261)		**4.698 (*p* = 0.033)**	**15.675 (***p* < 0.001)	
LF/HF ratio	1.822 (*p* = 0.179)	0.098 (*p* = 0.755)		0.249 (*p* = 0.619)	2.765 (*p* = 0.100)	

Bold values indicate a significant finding; *F*-statistics are results of type III test of fixed effects; parameter estimates of significant findings are provided in the text.

FC, face cooling; HF, high frequency; HR, heart rate; LF, low frequency; MAP, mean arterial pressure; PPCS, persisting postconcussive symptoms; RMSSD, root mean square of successive differences.

**FIG. 3.**
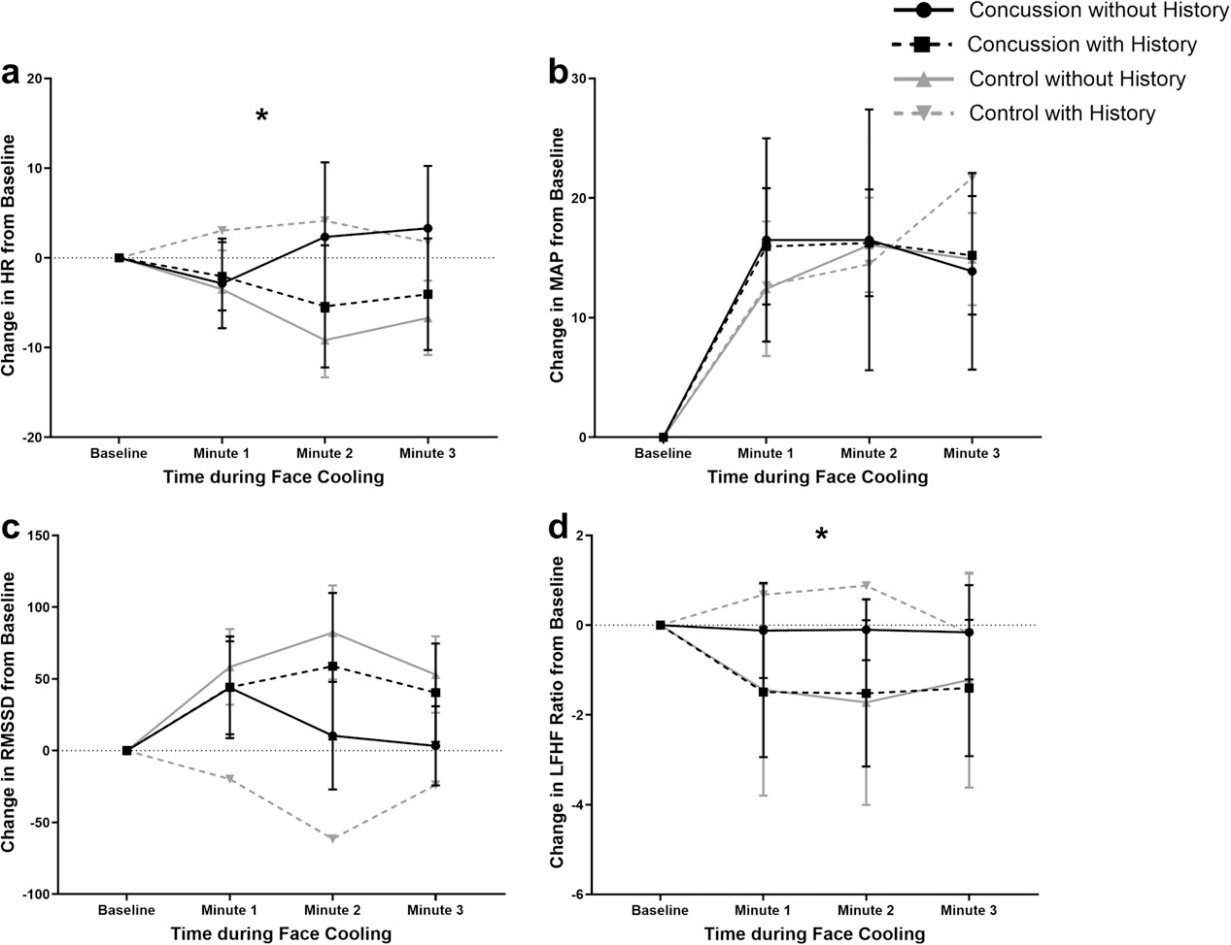
Groupwise means for ΔHR, ΔMAP, ΔRMSSD, and ΔLF/HF ratio during FC at Visit 1 stratified by history of concussion. *A significant difference between concussion group and history of concussion group overtime during FC at Visit 1. FC, face cooling; HF, high frequency; HR, heart rate; LF, low frequency; MAP, mean arterial pressure; RMSSD, root mean square of the successive differences.

Figure 3a–d presents the groupwise means for ΔHR, ΔMAP, ΔRMSSD, and ΔLF/HF ratio during FC at Visit 1 after stratifying the concussed and control participants by the incidence of prior concussion. Table 4 presents the results of the exploratory analysis that controlled concussion history for each of the main outcome variables. The effect of concussion and FC minute was not significant for any variable, and the effect of concussion history and FC minute was significant only for RMSSD. The interaction term of concussion, concussion history, and FC minute was significant for HR (*p* = 0.005) and RMSSD (*p* < 0.001). Analyzing the groupwise parameters estimates for HR revealed that concussed participants who did not have a history of concussion (*n* = 7, HR change/min = −0.190 [−2.326, 1.947], *p* = 0.859) and controls with a history of concussion (*n* = 2, HR change/min = 0.758 [−2.892, 4.408], *p* = 0.680) had blunted responses to FC. These responses were not significantly different from baseline values. However, concussed participants with a history of concussion (*n* = 17, HR change/min = −2.274 [−3.976, −0.572], *p* = 0.011) and controls without a history of a concussion (*n* = 22, HR change/min = −3.471 [−5.047, −1.895], *p* < 0.001) had a significant response. Groupwise parameter estimates for RMSSD are provided in Supplementary Table S1.

Table 5 presents the ICC of HR, MAP, RMSSD, and LF/HF ratio between visits for concussed participants with normal recovery, concussed participants with delayed recovery, and controls. The ICC for the 2-min change in controls is the true measure of retest reliability since concussed participants were symptomatic at Visit 1 but not at Visit 2. The 2-min HR, MAP, and RMSSD changes were reliable measures, whereas the LF/HF ratio was not. When comparing ICC coefficients, the HR and MAP responses were more reliable than RMSSD.

**Table 5.** Retest Reliability of the FC Response Between Visits 1 and 2

	Controls	Normal recovery	Delayed recovery
Baseline, Cronbach’s alpha (95% CI)			
HR	0.423 (−0.543, 0.784)	0.836 (0.357, 0.959)	0.420 (−0.610, 0.849)
MAP	**0.670 (0.144, 0.873)**	0.162 (−2.376, 0.792)	**0.746 (−0.128, 0.943)**
RMSSD	**0.917 (0.784, 0.968)**	**0.733 (−0.073, 0.934)**	−0.008 (−3.467, 0.773)
LF/HF ratio	0.461 (−0.400, 0.792)	0.328 (−1.705, 0.833)	0.136 (−2.830, 0.805)
Minute 2, Cronbach’s alpha (95% CI)			
HR	**0.678 (0.077, 0.887)**	**0.876 (0.502, 0.969)**	**0.815 (0.182, 0.958)**
MAP	**0.701 (0.143, 0.895)**	−0.020 (−3.105, 0.747)	**0.928 (0.682, 0.984)**
RMSSD	**0.669 (0.142, 0.873)**	**0.950 (0.799, 0.988)**	0.115 (−2.925, 0.800)
LF/HF ratio	−0.098 (−1.849, 0.577)	**0.786 (0.137, 0.947)**	0.205 (−2.524, 0.821)
Change from baseline to minute 2, Cronbach’s alpha (95% CI)			
HR	**0.828 (0.554, 0.934)**	0.657 (−0.383, 0.915)	0.321 (−2.012, 0.847)
MAP	**0.815 (0.449, 0.938)**	0.612 (−0.560, 0.904)	**0.831 (0.249, 0.962)**
RMSSD	**0.694 (0.204, 0.882)**	0.744 (−0.032, 0.936)	−1.621 (−10.621, 0.409)
LF/HF ratio	0.436 (−0.464, 0.734)	0.279 (−1.905, 0.821)	−0.040 (−3.608, 0.766)

Bold values indicate a significant finding. Values are Cronbach’s α with 95% CI. Negative values are theoretically impossible and should be interpreted as zero reliability.^28^

CI, confidence index; FC, face cooling; HF, high frequency; HR, heart rate; LF, low frequency; MAP, mean arterial pressure; RMSSD, root mean square of successive differences.

## Discussion

This study compared the hemodynamic and autonomic responses to FC in adolescents with SRC within 10 days of injury and after clinical recovery and compared them to age and sex-matched controls. Contrary to our primary hypothesis and pilot data,^17^ adolescents with acute SRC did not have a significant blunting of the autonomic response to FC. This may be due to the effect of age, concussion history, or because we did not meet our desired sample size. The pilot study included collegiate athletes, whereas the current study included adolescents. Adolescents have longer times between successive heartbeats and increased variability in resting HRV compared with young adults,^29^ which may explain differences in autonomic responses to FC. However, after concussed participants had recovered and returned to sport, they had a blunted HR response to FC compared with control participants. This is consistent with the observation that a variety of physiological parameters do not appear to normalize after concussion and persist beyond clinical recovery.^30^ The clinical relevance of whether this represents ongoing pathophysiology or adaptation is unclear.

### Typical recovery versus PPCS

As part of our confounder analysis, we stratified concussed participants into those who had typical (recovery <28 days since injury) and delayed recovery (PPCS, ≥28 days). We analyzed data from the initial visit and after they had recovered (Supplementary Figure S1.). There were no differences between groups at either time, suggesting that FC may not be able to prognosticate the risk for delayed recovery from SRC.^31^ Surprisingly, at the initial assessment, participants who would go on to have delayed recovery were more similar to the healthy adolescents than those who had a typical recovery. However, participants with delayed recovery had a larger variance (greater standard deviation and range), suggesting that participants with delayed recovery had a blunted response whereas others had an exaggerated one. Concussions are heterogeneous, and clinical classifications,^32^ have been proposed to stratify patients based on their predominant symptoms and signs.^33^ The physiological/autonomic concussion subtype is defined by reduced aerobic exercise performance and orthostatic intolerance on physical examination.^34^ Patients within this subtype typically recover faster than patients classified into the vestibular and oculomotor,^35^ or mood and cognition-related,^36–38^ subtypes. Hence, it is possible that blunting of the FC response is associated primarily with the physiological/autonomic subtype of concussion since all of our concussion participants met the inclusion criteria of concussion-related exercise tolerance. We calculated Cronbach’s alpha between visits in both concussion groups (normal and delayed recovery, Table 5). The 2-min HRV response did not have good retest reliability (except for MAP in the delayed recovery group), which suggests that the FC response changes from injury to recovery.

### History of concussion

Within our exploratory analysis of the effects of concussion history, we found that controls with a history of concussion demonstrated a blunted FC response compared with concussion naïve control participants. This corroborates the findings of another study on healthy high school and collegiate athletes with a remote (1–3 years prior) history of concussion demonstrating a blunted response compared with healthy athletes without any history of concussion.^27^ Nevertheless, this data must be interpreted cautiously because there were only two healthy participants (∼8% of the controls) with a history of concussion.^39^ In addition, HRV metrics are influenced by sex and body size,^11,40^ and the two healthy participants with a history of concussion were an 88 kg male and a 46 kg female. We do not know when, or if, a blunted response to FC returns to normal after an athlete has recovered from a concussion. We recommend that future studies that compare athletes with and without SRC account for concussion history in their analyses.

Our data also found that concussed adolescents with a history of concussion had similar responses to FC compared with concussion naïve controls (i.e., a significant reduction in HR and an increase in RMSSD and MAP). However, the concussed adolescents without a history of concussion had a blunted FC response. On visual inspection of Figure 3, those with a blunted response had an appropriate HR and RMSSD response through the first minute but started to return to baseline levels by the second minute. Concussed participants with a history of concussion and control participants without a history of concussion demonstrated a peak response by the second minute that started to return to baseline by the third minute. This delay in the return to baseline values by the third minute is likely due to a reactive sympathetic activation.^15^ Therefore, those with a blunted response to FC either had diminished parasympathetic responses or earlier sympathetic activation. It is unclear why this was not observed in concussed participants who had experienced a concussion prior to this study. Therefore, we suggest that future studies aim to better understand this.

### Retest reliability

Excluding the LF/HF ratio, the HR, MAP, and RMSSD responses to FC were reliable in controls. The reliability for HR and MAP was strong (ICC range 0.75–0.9), whereas the reliability for RMSSD was moderate (range 0.5–0.75).^41^ Previous literature recommends analyzing HRV from a 5-min recording instead of a 1-min recording to increase the reliability of readings.^42^ We were unable to collect longer recordings due to the brevity of the FC test. Therefore, we recommend that subsequent studies that utilize FC should focus on hemodynamic variables (i.e., HR and MAP) rather than HRV due to concerns about reproducibility and the influence of recording duration.^42^

### Limitations

Cardiovascular parameters, especially HRV metrics, are influenced by multiple conditions, including stress, age, sex, body size, athletic fitness, hormone variation, and menstrual cycle phase.^43,44^ Sex and age were similar between groups; however, the other variables were not controlled for and were assumed to affect each group randomly. Another limitation of this study is that concussed participants who developed PPCS took months to recover, whereas the ones in the typical recovery group resumed sports within a few weeks. Those who took longer to recover may have been aerobically deconditioned,^45,46^ by their postrecovery assessment even though they had returned to sport, which may have affected the HRV analysis.

## Conclusion

Adolescent athletes within 10 days of SRC did not demonstrate a significantly blunted parasympathetic response to FC, which differs from prior reports on acutely concussed collegiate athletes. While not different from nonconcussed athletes, the response to FC did not return to initial values after participants had clinically recovered from their SRC. In addition, autonomic responses to FC were unable to differentiate concussed adolescents who would go on to have delayed recovery from those who would have normal recovery times. Our data also found that concussed adolescents without a history of prior concussion had a blunted parasympathetic response to FC whereas those with a concussion history had similar responses to controls. Therefore, it is crucial that future studies on the role of the ANS in concussion control for concussion history in addition to sex, age, and fitness level. Future studies are also warranted to understand the chronic adaptations in the brain that occur following concussion.

## Transparency, Rigor, and Reproducibility Summary

The University at Buffalo institutional review board registered and approved this experimental case-control study with the protocol number STUDY00000092. The study approval was obtained in January 2016. After training personnel and pilot testing the protocol, we began recruitment in October 2016. We had recruited 71% of our desired sample before the pandemic occurred and our exercise physiology lab suspended all operations for ∼1 year. During this time, large changes in lab personnel also occurred. We began recruiting again in the middle of 2021, but eligible patients did not consent to participate, often citing COVID-19 exposure as too significant of a risk to consider participation in a voluntary study. Therefore, our recruitment plateaued, and we ended the study before meeting the desired sample size. The research team was then unblinded to perform formal data analysis and interpretation. Comparison between concussed and controls and between early and delayed recovery were *a priori* aims, but the inclusion of concussion history was based on a study published in 2020,^27^ that found blunting of the ANS more than 1 year after recovering from SRC. Although we did not reach our sample size or perform statistical tests of equivalence, our interpretation that an acute SRC does not cause blunting of the ANS is based on the negligible difference in 2-min change in RMSSD (1 ms^−2^ with a standard deviation of ∼90, corresponding to an effect size of 0.01) between acute SRC with history of concussion and healthy controls without.

## Abbreviations Used

ANS: autonomic nervous system
BP: blood pressure
CO: cardiac output
EKG: electrocardiogram
FC: face cooling
HF: high frequency
HR: heart rate
HRV: heart rate variability
ICC: intraclass correlation coefficient
LF: low frequency
MAP: mean arterial pressure
PPCS: persisting post concussion symptoms
RMSSD: root mean squared of successive differences
RTP: return to play
RRI: r-r interval
SV: stroke volume
SRC: sport-related concussion
