# Supplementary material for: Parasympathetic Responses to Face Cooling in Adolescents with Sport-Related Concussion and After Clinical Recovery
Source: Neurotrauma Rep. 2025 Jan 23;6(1):93–105. doi: 10.1089/neur.2024.0138 (PMC11839524; doi:10.1089/neur.2024.0138)

Supplement file for “**Parasympathetic Response to Face Cooling in Adolescents with Acute Sport-related Concussion and after Clinical Recovery**”

**Figure e1.** Groupwise means for ΔHR, ΔMAP, ΔRMSSD and ΔLF/HF ratio during FC at Visit 2 stratified by recovery time.


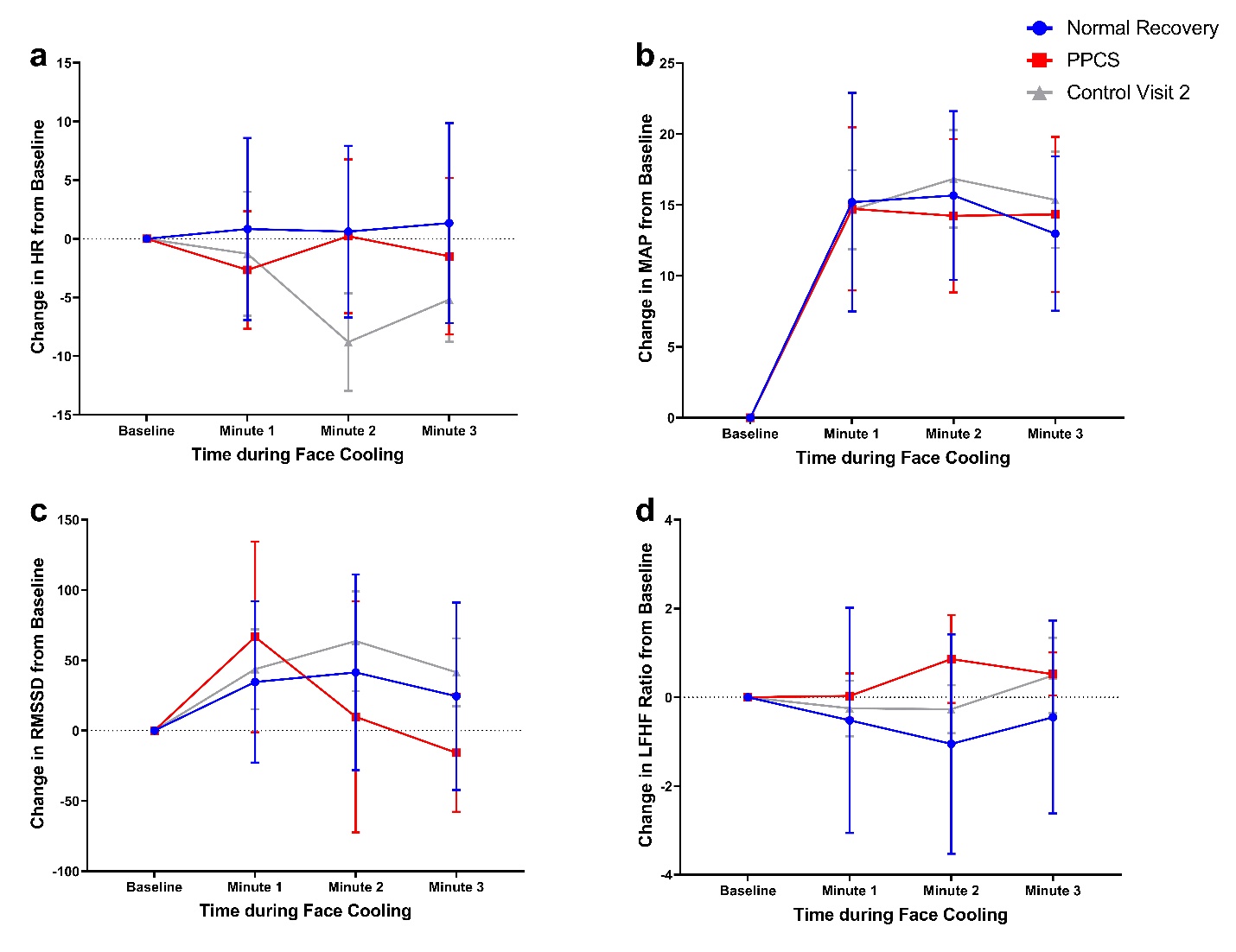

Supplement: Supplementary Figure S1 [file neur.2024.0138_supp_figure_s1.docx]
