# Supplementary material for: Parasympathetic Responses to Face Cooling in Adolescents with Sport-Related Concussion and After Clinical Recovery
Source: Neurotrauma Rep. 2025 Jan 23;6(1):93–105. doi: 10.1089/neur.2024.0138 (PMC11839524; doi:10.1089/neur.2024.0138)
Supplement: Supplementary Table S1 [file neur.2024.0138_supp_table_s1.docx]

**Table e1.** Parameter estimates for RMSSD change/minute for Concussion History exploratory analysis

| **Group** | **Estimate** | **Std. Error** | ***t*** | ***p*-value** | **Lower 95%** | **Upper 95%** |
| --- | --- | --- | --- | --- | --- | --- |
| Concussion without History*FC Minute | 12.968 | 7.517 | 1.725 | 0.089 | -2.006 | 27.942 |
| Concussion with History*FC Minute | 26.539 | 5.882 | 4.512 | <0.001 | 14.734 | 38.344 |
| Control without History*FC Minute | 34.396 | 5.349 | 6.430 | <0.001 | 23.592 | 45.200 |
| Control with History*FC Minute | -12.093 | 12.824 | -0.943 | 0.348 | -37.605 | 13.419 |
| a. Timepoint = Visit 1 | | | | | | |
| b. Dependent Variable: RMSSD | | | | | | |
